# Genetic assemblage of *Sarcocystis* spp. in Malaysian snakes

**DOI:** 10.1186/1756-3305-6-257

**Published:** 2013-09-09

**Authors:** Yee Ling Lau, Phooi Yee Chang, Vellayan Subramaniam, Yit Han Ng, Rohela Mahmud, Arine Fadzlun Ahmad, Mun Yik Fong

**Affiliations:** 1 Department of Parasitology, Faculty of Medicine, University of Malaya, 50603 Kuala Lumpur, Malaysia; 2 Faculty of Pharmacy, Universiti Teknologi MARA, 43200 Puncak Alam, Selangor, Malaysia

**Keywords:** Definitive host, *Sarcocystis*, Snake, 18S rDNA, Phylogenetic analysis

## Abstract

**Background:**

*Sarcocystis* species are protozoan parasites with a wide host range including snakes. Although there were several reports of *Sarcocytis* species in snakes, their distribution and prevalence are still not fully explored.

**Methods:**

In this study, fecal specimens of several snake species in Malaysia were examined for the presence of *Sarcocystis* by PCR of 18S rDNA sequence. Microscopy examination of the fecal specimens for sporocysts was not carried as it was difficult to determine the species of the infecting *Sarcocystis*.

**Results:**

Of the 28 snake fecal specimens, 7 were positive by PCR. BLASTn and phylogenetic analyses of the amplified 18S rDNA sequences revealed the snakes were infected with either *S. nesbitti*, *S. singaporensis*, *S. zuoi* or undefined *Sarcocystis* species.

**Conclusion:**

This study is the first to report *Sarcocystis* infection in a cobra, and *S. nesbitti* in a reticulated python.

## Background

*Sarcocystis* is an intracellular protozoan belonging to the family Sarcocystidae and is closely related to medically important protozoa, such as *Toxoplasma gondii* and *Neospora caninum*. Its life cycle involves intermediate and definitive hosts and is based on predator–prey relationships [1]. Asexual stages develop in intermediate hosts, ultimately resulting in the formation of intramuscular cysts (Sarcocysts). Following ingestion of tissue containing sarcocysts by a definitive host, the sexual stages of the life cycle are initiated in the intestine, producing oocysts that are excreted in feces [1]. Sarcocystosis is a zoonotic disease that is found mostly in tropical and sub-tropical countries, which usually affects wild and domestic animals, such as cows and pigs [2]. Notably, humans may serve as definitive hosts for *Sarcocystis hominis* and *Sarcocystis suihominis* after consuming raw, infected meat from cattle and pigs, respectively. However, humans can also be intermediate hosts for some *Sarcocystis* species of unknown origin. In such cases, humans can be infected through food or drinks that are contaminated with feces containing sporocyst from infected animals. In fact, a study identified sarcocystosis antibodies in 20% of Malaysians [3], and tissue autopsies showed 21 out of 100 individuals contained sarcocysts [4]. In December 2011, the surveillance program of the International Society of Travel Medicine and Centers for Disease Control and Prevention (CDC) reported an outbreak of acute muscular sarcocystosis, which involved 32 travellers to Tioman Island off the coast of peninsular Malaysia [5]; however, the *Sarcocystis* species involved was not identified.

Muscular sarcocystosis can be diagnosed through microscopic examination of histologic sections stained with hematoxylin and eosin. Nevertheless, technical variability in staining and tissue sectioning procedures might be anticipated [6]. Additionally, the ultrastructure of many species is inadequately described. Thus, definitive diagnosis of sarcocystosis requires identification of sporocysts in feces. However, the sporocysts of different species are similar in size and shape, making species identification almost impossible by microscopy. Therefore, sequencing of the small subunit ribosomal RNA (18S rRNA) gene was introduced as an ideal means for species-specific detection [7]. In fact, this gene contains hypervariable regions interspersed within highly conserved DNA sequences, making it ideal for differentiation between species. For this reason, the 18S rRNA gene has been frequently used in phylogenetic analyses of species within the Apicomplexa [8-14]. Moreover, the large number of 18S rRNA gene sequences available for different *Sarcocystis* species should enable construction of an informative phylogenetic tree of the Sarcocystidae. Indeed, the utility of 18S rRNA gene sequencing for differentiation of *Sarcocystis* species and for phylogenetic studies has been demonstrated by several researchers [15-18].

SP Kan and R Pathmanathan [19] have highlighted five well-defined (complete life cycle) *Sarcocystis* species in Malaysia. Notably, it was identified that three out of five of these *Sarcocystis* species (i.e., *S. singaporensis*, *S. villivillosus*, and *S. zamani*) circulate between pythons (definitive hosts) and rats (intermediate hosts) [20], indicating the high diversity of *Sarcocystis* species infecting snakes. During their research in Thailand, T Jakel, Y Khoprasert, I Sorger, D Kliemt, V Seehabutr, K Suasa-ard and S Hongnark [21] confirmed that *Broghammerus/Python reticulatus*[22] served as a suitable definitive host for *S. singaporensis* and *S. zamani*. Furthermore, in other countries, P Daszak and A Cunningham [23] as well as Šlapeta and his collaborators (2003) discovered *Sarcocystis* species parasitizing a bullsnake (*Pituophis melanoleucus sayi*) and African tree vipers (*Atheris* spp. and *Bitis* spp.), respectively.

Here, we have performed phylogenetic analyses based on sequencing of the 18S rRNA gene in order to determine evolutionary relationships between *Sarcocystis* species isolated from Malaysian snakes.

## Methods

### Sample collection

Fecal samples of snake were collected from Perak (Taiping Zoo and Pangkor Island), Kelantan (Kuala Kerai), Kedah (Langkawi Wildlife Park) and from isolated private snake owners (Sri Gombak and Cheras), Malaysia. A total of 28 snakes were caught, including 14 reticulated python (*Broghammerus/Python reticulates*), 3 Malayan Brown Pit Viper (*Ovophis convictus*), 2 King cobra (*Ophiophagus hannah*), 1 Monocled cobra (*Naja kaouthia*), 1 Wagler’s keeled green pit viper (*Tropidolae muswagleri*), 1 Malayan keeled rat snake (*Ptyas carinata*), 1 Albino python (*Python bivittatus*), 1 Boa python (*Python regius* subspecies), 1 Ball python (*Python regius*), 1 Mangrove snake (*Boiga dendrophila melanota*), 1 Mexican black king snake (*Lampropeltis getula nigrita*) and 1 King snake (*Lampropeltis* sp.). The snakes consumed similar diets such as rats and frogs. The fecal samples were stored in 2.5% potassium dichromate and kept at 4°C for further use. This research was carried out with the approval by the University Malaya Medical Ethics Committee (Ref no. 920.16).

### Sample processing and examination

The preserved samples were examined under a microscope. Fecal samples were concentrated by formalin-ether concentration and filtered to remove the course debris followed by microscopic examination.

### DNA isolation

Total DNA from fecal samples was isolated using the PowerSoil® DNA Isolation Kit (MO BIO Laboratories) according to the manufacturer’s protocol. Briefly, 0.25 g of the fecal sample was lysed by adding the solution containing SDS (Solution C1). The non-DNA organic and inorganic materials in the lysed sample were removed by adding Solution C2 and Solution C3. Following centrifugation, the supernatant was mixed with high salt solution (Solution C4) and loaded into the Spin Filter to allow binding of DNA to the silica membrane. The bound DNA was cleaned with ethanol wash solution (Solution C5) and followed by elution of the DNA by using 30 μl elution buffer (Solution 6).

### Polymerase chain reaction

The 18S rRNA gene was amplified by nested PCR, using primer 1 L (5′-CCATGCATGTCTAAGTATAAGC-3′) and primer 1H (5′-TATCCCCATCACGATGCATAC-3′) in the primary reaction, followed by primer 3 L (5′-CTAGTGATTGGAATGATGGG-3′) and primer 2H (5′-ACCTGTTATTGCCTCAAACTTC-3′) in the secondary reaction [18]. Four μl of the DNA template was used in a 25 μl PCR reaction, with the following reaction conditions: 35 mM Tris–HCl, pH 9.0, 25 mM KCl, 3.5 mM MgCl2, 5 pmoles of each primer, 1 mM dNTPs, and 1 U *Taq* polymerase (Promega). The PCR was performed as follows: 95°C for 2 min, followed by 35 cycles of 94°C for 40 sec, 50°C for 30 sec, 72°C for 1.5 min, followed by 72°C for 6 min [18]. The product was separated by agarose gel electrophoresis.

### Cloning and sequencing

Amplicons were cloned using pGEM®-T Vector System (Promega) and positive clones from each amplicon were sequenced in both directions by using M13 universal primers. Sequence analysis was performed using the BioEdit software.

### Nucleotide search and phylogenetic analysis

Each 18S rRNA gene sequence was searched for match in the GenBank nucleotide database using the Basic Local Alignment Search Tool (BLASTn). Two to three sequences were chosen to represent each amplicon in phylogenetic analysis. The sequences were then compared and aligned with 18S rRNA gene sequences of *Sarcocystis* species available in GenBank. Multiple sequence alignment was carried out using ClustalW. A phylogenetic tree was constructed based on the 18S rRNA gene sequences by Neighbor-Joining method (bootstrap = 1000) available in MEGA4 [24]. The 18S rRNA gene sequence of *Eimeria tennella* was used as outgroup.

## Result

**Figure 1:**
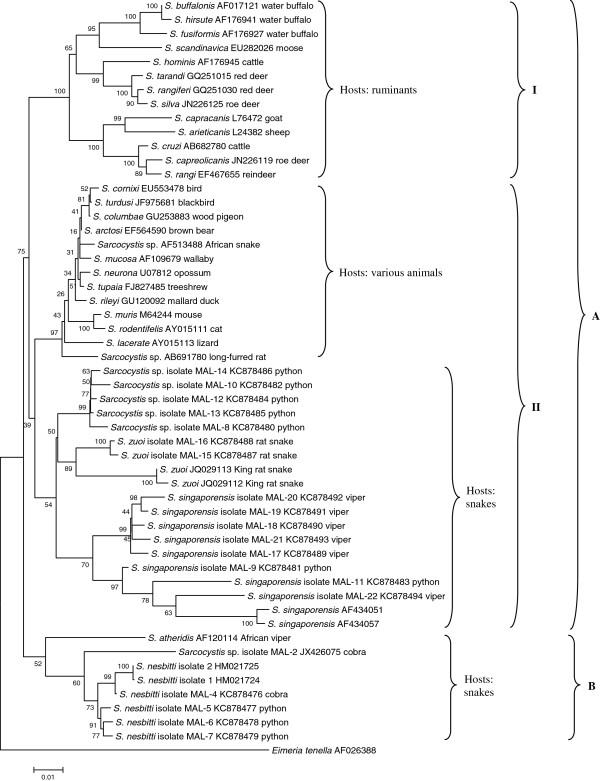
**Phylogenetic tree based on 18S rRNA sequences of *Sarcocystis *species.** The tree is constructed using the Neighbour Joining method. The percentage of replicate trees in which the associated sequences clustered together in the bootstrap test (1000 replicates) are shown next to the branches [27]. The tree is drawn to scale, with branch lengths in the same units as those of the evolutionary distances used to infer the phylogenetic tree. The phylogenetic analysis was conducted using MEGA4 [24].

In this study, 28 snake fecal samples were obtained but no oocyst/sarcocyst was found in the stools using microscopy. Seven snakes (25%) were found to be positive for *Sarcocystis* 18S rRNA by PCR. The 7 infected snakes included 4 reticulated pythons from Langkawi Wildlife Park; 1 Malayan brown pit viper from Pangkor; 1 Monocled cobra from Pangkor and 1 Malayan keeled rat snake from Kuala Kerai (Table 1). Amplicons from the PCR reaction were cloned into pGEM®-T and sequenced. Twenty 18S rRNA sequences were obtained and each sequence was searched for match in the GenBank database using BLASTn. Once the species was determined, a phylogenetic tree was constructed based on these 20 sequences plus 34 other *Sarcocystis* 18S rRNA sequences (Figure 1). The phylogenetic tree shows two major clades (A, B) of *Sarcocystis*. Clade A is divided into two subclades (I, II). Subclade I consisted of species that infect ruminants. Subclade II is further divided into two groups, one consisting of species whose hosts are animals such as birds, opossum, treeshrew and lizard. The other group consisted of *Sarcocystis* species in which snakes (python, ratsnake, viper) are the definitive hosts, such as *S. singaporensis* and *S. zuoi*. Interestingly, clade B contains only one group. The members of this group, *S. nesbittii* and *S. atheridis*, use snakes (python, cobra, viper) as definitive hosts. The *Sarcocystis* detected in the snake fecal samples of our study are distributed in both clades A and B.

**Table 1:** **Summary of *Sarcocystis *species found in infected snakes**

**Snake taxa *species***	**State**	**Number of snake**	**Clone name**	**Sarcocystis species**	**Possible definitive host**	**Possible intermediate host**	**References**
Cobra *Naja kaouthia*	Kuala Kerai	1	MAL-2, MAL-4	*S. nesbitti*	Snake	Monkeys	[25,26]
						(*Macaca mulatta*)	
						(*Macaca fascicularis*)	
						(*Cercocebus atys*)	
						(*Papio papionis*)	
Reticulated python *Braghammerus reticulatus* (Previously known as *Phyton reticulatus*)	Langkawi Wildlife Park	2	MAL-9, MAL-11	*S. singaporensis*	Reticulated python	Genera *Rattus* and *Bandicota*	[21]
					(*Broghammerus reticulatus*)		
		1	MAL-5, MAL-6, MAL-7	*S. nesbitti*	Undefined	Monkeys	[25,26]
						(*Macaca mulatta*)	
						(*Macaca fascicularis*)	
						(*Cercocebus atys*)	
						(*Papio papionis*)	
		1	MAL-8, MAL-10, MAL-12, MAL-13, MAL-14	*Sarcocystis* sp.	-	-	-
Malayan Brown Pit Viper *Ovophis convictus*	Pangkor Island	1	MAL-17 to MAL-22	*S. singaporensis*	Reticulated python	Genera *Rattus* and *Bandicota*	[21]
					(*Broghammerus reticulatus*)		
Malayan Keeled rat snake *Ptyas carinata*	Kuala Kerai	1	MAL-15, MAL-16	*S. zuoi*	King rat snakes	Norway rats	[16]
					(*Elaphe carinata*)	(*Rattus norvegicus*)	

## Discussion

This is the first report identifying infection of a monocled cobra (*N. kaouthia*) with *Sarcocystis* species. In addition, phylogenetic analysis based on sequencing of the 18S rRNA gene indicated that the *Sarcocystis* species isolated from the monocled cobra was most closely related to *S. nesbitti*, which was first discovered by [25] in rhesus monkey muscle. Mandour’s work had been supported years later with the proof of light and transmission electron micrograph [26]. In 2011, Tian and his collaborators hypothesized that a snake might serve as the definitive host for *S. nesbitti* based on phylogenetic analyses [17]. This was due to the fact that *S. nesbitti* closely resembled certain *Sarcocystis* species that cycle between rodent intermediates and snakes (e.g., African tree vipers) [8]. Moreover, in our study, *S. nesbitti* was also found to infect reticulated pythons from Langkawi. Our findings have thus confirmed that snakes are likely to be the definitive host for *S. nesbitti*.

Four infected reticulated pythons were examined in this study: two with *S. singaporensis*, one with *S. nesbitti*, and the last with an unknown *Sarcocystis* species. Similarly in other studies, boid snake (*Broghammerus/Python reticulatus*) has shown to be the natural definitive host of *S. singaporensis*. *S. singaporensis* develops sexually in the intestine of the snake to produce sporocysts, which are released in the faces [20]. Suitable intermediate hosts for *S. singaporensis* include all of *Rattus* and *Bandicota* species as well as *Nesokia indica*[20,28-30]. Furthermore, *S. singaporensis* is endemic in Southeast Asia [31-34], and considered to be highly pathogenic. In fact, it can be lethal due to extensive development of schizonts in rat endothelial cells [28]. As a result, it could potentially be used as a biological agent for controlling wild rodent populations in non-native environments [35].

Previous studies have reported vipers to be the definitive hosts for a few species of *Sarcocystis*, including *S. atheridis* in Nitsche’s bush viper (*Atheris nitschei*) [36], *S. hoarensis* in *Bitis arientans*[37], and *Sarcocystis muriviperae* in Palestine viper (*Vipera palestinae*) [37]. In the present study, Malayan brown pit viper was infected with *S. singaporensis*.

*S. zuoi* was detected in Malayan keeled rat snake. Indeed, *S. zuoi* is commonly found in Norway rats (*Rattus norvegicus*), and transmission experiments carried out in China have indicated that the king rat snake is the definitive host of *S. zuoi*[16].

So far, phylogenetic relationships among the majority of analyzed *Sarcocystis* species have suggested their coevolution with final rather than intermediate hosts [8]. Our results are consistent with this notion because all of the *Sarcocystis* species that we have isolated from snakes are related to *S. nesbitti*, *S. singaporensis*, and *S. zuoi*, which are *Sarcocystis* species using snakes as definitive hosts.

## Conclusion

We have demonstrated a high (25%) prevalence of *Sarcocystis* infection in snake populations in peninsular Malaysia. Taken together, we have identified snakes infected by *S. nesbitti*, *S. singaporensis*, *S. zuoi*, and some undefined species. These findings contribute to our phylogenetic understanding of the Sarcocystidae and provide valuable information to build upon for future research.

## Competing interests

The authors have no financial or personal relationship with other people or organizations that could inappropriately influence or bias this paper.

## Authors’ contributions

PYC carried out the experiment, YLL contributed most on manuscript writing. YHN helped in manuscript writing and editing, MYF helped on phylogenetic tree construction. RM and AA provided opinions and suggestions about this manuscript while SV supplied useful information about the snakes in South East Asia. All authors read and approved the final version of the manuscript.
